# Telerehabilitation from the perspective of patients and healthcare providers: A 3-year follow-up study

**DOI:** 10.3233/NRE-230385

**Published:** 2024-08-29

**Authors:** Lucia Willadino Braga, Sandro Barbosa Oliveira, Lígia Maria do Nascimento Souza

**Affiliations:** SARAH Network of Rehabilitation Hospitals, Brasilia, Brazil

**Keywords:** Telemedicine, telerehabilitation, rehabilitation, telehealth, patient satisfaction

## Abstract

**BACKGROUND:**

It is important to investigate satisfaction and perception of the effectiveness of telerehabilitation and its outcomes post-COVID-19 pandemic.

**OBJECTIVE:**

Evaluate the patients’ and healthcare providers’ level of satisfaction with telerehabilitation and perception of its efficacy and describe how it became an established resource in a network of rehabilitation hospitals post-pandemic.

**METHODS:**

The online survey about their experience with telerehabilitation was completed by 2,755 patients (322 new patients and 2,433 existing patients), and 668 providers from 26 different specialties.

**RESULTS:**

Most patients and providers rated remote care as effective. There were no differences in scores between existing patients and new patients and the majority reported that their expectations were met. Most patients described their remote consults as good as or better than in-person, while providers mostly preferred in-person sessions. Despite most modalities having returned to in-person practice, there is still a significant percentage of telerehabilitation consults.

**CONCLUSION:**

Three years after the start of the pandemic, despite a return to in-person treatment, we see a telerehabilitation culture being constructed as a resource to supplement the rehabilitation process, with potential for establishing this model in a network of rehabilitation hospitals.

## 1 Introduction

Chronic neurological disorders of the brain, spinal cord, and locomotor system require life-long follow-up and management by interdisciplinary teams (Hart et al., 2018; Lavis & Goetz, 2019; Lindsay et al., 2020; Thomsen et al., 2019). With the COVID-19 outbreak, much of the world’s medical resources concentrated on patients with the coronavirus. As a result, elective and rehabilitation procedures were suspended in many countries to shield patients with chronic illness, compromised immunity or comorbidities. There was an urgent need for a system to continue offering interdisciplinary health services for this population (Chaler et al., 2020; López-Dolado & Gil-Agudo, 2020; Veerapandiyan et al., 2020). Additionally, the social isolation caused by the pandemic created many challenges for the providers and teams working within the traditional, in-person models of rehabilitation.

Within this context, telerehabilitation emerged as a valuable resource for the ongoing treatment of these patients. Technological advancements, such as videoconferencing, highspeed internet, and more affordable computers, apps, and devices, have contributed significantly to the incorporation of IT-based tools in rehabilitation (Torsney, 2003). Telerehabilitation rapidly expanded to become routinely used world-wide as an alternative to in-person services (Hincapié et al., 2020).

Before the COVID-19 pandemic, remote delivery of rehabilitation services was quickly expanding, particularly with patients who could not attend therapy in person due to mobility or transportation issues (López et al., 2020; Torsney, 2003). Telerehabilitation was already shown to be as efficient and effective as conventional rehabilitation methods in neurological rehabilitation, cardiac rehabilitation, follow-up of individuals with spinal cord injuries, rehabilitation for speech-language impairments, and rehabilitation for varied patient populations (Kairy et al., 2009). Systematic reviews showed low-to-moderate evidence that telerehabilitation services were not inferior to in-person treatment for stroke patients. Telerehabilitation interventions can improve deficiencies, disabilities, and quality of life of stroke survivors, while helping to reduce depression and increase the wellbeing of their caregivers (Laver et al., 2020, Tchero et al., 2018).

Since the arrival of COVID-19, various rehabilitation subspecialties have come to rely on telerehabilitation, or aspects of remote therapy, as a delivery mode for treatment (Hincapié et al., 2020). A study by Chang et al. (2021) showed that remote monitoring of patients after they were discharged from hospital stroke units during the pandemic was successful, indicating that telerehabilitation is accessible and effective in patients with neurovascular disorders. Some studies report that telerehabilitation is comparable or even superior to conventional treatments in terms of cognitive and functional results in patients with neurological impairment, such as stroke or multiple sclerosis (Cacciante et al., 2022; Sharififar et al., 2023). Solomon et al. (2022) noted the benefits of telerehabilitation for patients with spinal cord injury. Other studies showed promising results in a variety of conditions, such as osteoarthritis, back pain, and knee and hip replacements (Bhuva et al., 2020; Muñoz-Tomás et al., 2023; Seron et al., 2021; Suso-Martí et al., 2021). Furthermore, telemedicine has been well received in other areas of healthcare, such as sports medicine (Tenforde et al., 2020) and pediatric rehabilitation (Camden & Silva, 2021; Hsu et al., 2021), especially during the COVID-19 crisis. Nevertheless, challenges persist, particularly in neuropsychology (Bilder et al., 2020; Hewitt et al., 2020; Hewitt & Loring, 2020), with concerns about assessments conducted remotely. There have been, however, studies indicating that telemedicine can be effective even in this field (Crivelli et al., 2022; Hammers et al., 2020; Wells et al., 2021), with proposals of a hybrid practice (Singh & Germine, 2021). For patients with long-term COVID-19, telerehabilitation has been an effective strategy for relieving symptoms, enhancing quality of life (Bernal-Utrera et al., 2022; Killingback et al., 2023; Valverde-Martínez et al., 2023) and improving functional capacity (Seid et al., 2022).

A study exploring the implementation of telemedicine in a clinic for physical therapy and spinal rehabilitation during COVID-19 showed that 64.5% of patients preferred telemedicine to in-person consults (Bhuva et al., 2020). Furthermore, telerehabilitation presents higher levels of satisfaction and adhesion among patients, with scores equal to traditional rehabilitation.

Growing evidence supports the viability and efficacy of remote healthcare (Aquino et al., 2021; Iodice et al., 2021; Maresca et al., 2020; Wahezi et al., 2020). Heiskanen et al. (2021) investigated the degree to which telerehabilitation was accepted among different rehabilitation professionals during the COVID-19 pandemic. Results showed that 52% of the therapists who participated in this study used telerehabilitation for most or all their patients during the first wave of COVID-19. Of the professionals who engaged in tele-rehabilitation during the pandemic, 46% planned to continue using it regularly after the pandemic.

Studies also address concerns about the economic aspects of telerehabilitation. For example, a systematic review by Grigorovich et al. (2022) formally documented the cost/effectiveness of at-home tele-rehabilitation versus in-person rehabilitation. The authors concluded that telerehabilitation may result in similar or lower costs than in-person rehabilitation; however, the impact on health-related quality of life is not yet clear. Most of the studies reviewed by Baffert et al. (2023) reported results for telerehabilitation that were more effective and less costly. Nizeyimana et al. (2022) focused on investigating the viability, cost, access to rehabilitation services, and implementation of telerehabilitation in countries of mid- to low socioeconomic status. They concluded that telerehabilitation could be a viable means of service delivery in countries of high, mid, and low socioeconomic status. Nevertheless, some barriers need to be surmounted, such as lack of knowledge and technical competence among the telerehabilitation providers and users of these remote services; limited resources; and secure platforms dedicated to telerehabilitation, among others.

Before the pandemic, the Brazilian Medical Federation did not permit remote delivery of medical services, or telemedicine. In March of 2020, the Ministry of Health began regulating telemedicine, with the aim of providing greater medical assistance and education and incentivizing research in healthcare. With a portion of the population confined to their homes, the use of interactive technology became an essential resource and safe alternative for protecting not only the patients’ health, but the providers’ as well. At that time, telemedicine helped ease the burden on healthcare institutions and contained the flow of people at a time when physical proximity was not safe. In addition, governmental regulation helped establish telemedicine as a way to more sustainably ensure the wider use of technology in healthcare (Morosini, 2021). Currently, various medical councils also regulate the use of telemedicine, in fields such as physical and occupational therapy (*Conselho Federal de Fisoterapia e Terapia Ocupacional*, 2020), psychology (*Conselho Federal de Psicologia*, 2020), speech therapy (*Conselho Federal de Fonoaudiologia*, 2020), nursing (*Conselho Federal de Enfermagem*, 2020) and nutrition (*Conselho Federal de Nutricionistas*, 2020).

Telemedicine is in the process of consolidation in Brazil. As telehealth is regulated in various healthcare fields, more and more providers offer remote treatment as an alternative to in-person care, thereby expanding access to medical care. The objective of this study is to assess the patients’ and the healthcare practitioners’ perception of effectiveness and satisfaction regarding telerehabilitation within a hospital network, across different specialties. It also aims to compare the responses among patients and practitioners, and to describe the establishment of telehealth as a supplementary resource in the rehabilitation process, three years after the start of the COVID-19 pandemic.

## 2 Material and methods

This study was conducted within a rehabilitation center in Brazil. The SARAH Network of Rehabilitation Hospitals comprises nine centers throughout Brazil and treats 1.8 million patients with chronic disabilities annually (https://www.sarah.br), free of charge. The patient population includes children and adults with brain disorders, spinal cord injury, rheumatic illness, chronic orthopedic disorders, and cancers of the spinal cord, brain, and bone.

### 2.1 Participants

In the first months of the COVID-19 pandemic, 19,578 patients with various chronic diseases who had been treated via telehealth at the SARAH Network of Rehabilitation Hospitals in Brazil, were invited to participate in a survey about their experience with telemedicine. A total of 2,755 patients completed the survey, of which 322 (12%) were new patients (first consult) and 2,433 (88%) were patients already in follow-up at SARAH Network Hospitals across Brazil.

Additionally, the rehabilitation practitioners were invited to assess their experience with telemedicine, and 668 providers completed the online survey to evaluate the patients’ experience from the professionals’ perspective. This sample of respondents included 26 different specialties: 14 medical specialties (i.e.: anesthesiology, clinical medicine, genetics, spinal cord injury, occupational medicine, neurosurgery, neurophysiology, oncology, orthopedics, pediatrics, psychiatry, neurological rehabilitation, rheumatology, and urology) and 12 paramedical specialties (i.e.: art education, social services, dance therapy, physical education, nursing, clinical pharmacy, speech therapy, nutrition, education, psychology, functional therapy, and occupation therapy). An e-invite was sent to patients and healthcare practitioners with a link to thesurvey.

In the early stages of the COVID-19 outbreak, we implemented wide-access synchronous telemedicine, via an extensive in-house technological infrastructure, to continue providing medical assistance, albeit remotely, to our patients during social isolation. Video and telephonic conferencing equipment was connected to medical charts and integrated with the hospital IT system. Since the start of the pandemic, more than 680,000 treatment sessions have been conducted.

Information about hospital location, diagnoses, practitioner specialty, and sociodemographic data (age and sex) was collected from the electronic medical charts of the respondents, whose ages ranged from less than a year to 96 years old.

Diagnoses were grouped into five categories: a) brain disorders (TBI, stroke, Parkinsons, dementia, cerebral palsy, and neuropathies); b) spinal cord injury (trauma to the spinal cord, spina bifida); c) oncology (tumors of the brain, bone, spinal cord, cartilage or connective tissue); d) rheumatic disease; e) chronic orthopedic disorders; f) other diagnoses (e.g. babies at risk, development disorders, patients undergoing diagnostic investigation).

Historical data on the use of telemedicine by different medical and paramedical specialties were obtained through the statistics department of the SARAH Network.

### 2.2 Instruments

Patients and rehabilitation practitioners answered a questionnaire containing four questions: (Q1) effectiveness of the consult; (Q2) comparison with in-person consults; (Q3) patient’s needs (new patients only); (Q4) overall satisfaction with the experience (see Supplementary Material for further details).

The perception of telerehabilitation effectiveness was evaluated according to the provider’s ability to listen to the patient’s complaints, address their concerns, and instruct and guide them through the rehabilitation process during theteleconsult.

Rehabilitation practitioners also assessed the feasibility (when applicable) of conducting medical activities during telemedicine appointments, including data collection (*N* = 667); physical-functional evaluation/exams (*N* = 552); application of formal tests and instruments (*N* = 430); guidance about the disease, activities, and care (*N* = 656); analysis/transmission of exam results and information (*N* = 565); prescription/proposal of new treatments (*N* = 551); and adjustments to the previously prescribed/proposed treatment plan (*N* = 576).

Those questions were developed through a focus group involving professionals from various specialties in the field of rehabilitation, with the assistance of a senior researcher.

A 5-level Likert Scale was used to rate and measure the responses. For Q1 and Q3, the “strongly disagree” to “strongly agree” scale was used. For comparing telemedicine to in-person consultations (Q2), we used degrees such as “much worse”; “somewhat worse”; “about the same”; “somewhat better” and “much better”. Overall satisfaction varied from “very poor” to “very good”. And the scale for procedures conducted during telerehabilitation, the range was “was not possible” to “was alwayspossible”.

The telerehabilitation percentage was calculated based on 2,906,112 outpatient appointments recorded from July 2020 to June 2023, encompassing both in-person and remote consultations.

### 2.3 Statistical analysis

Spearman’s rho correlation evaluated the association between the patient’s age and the variables on the questionnaire. Pearson’s Chi-squared test assessed the association between demographic/consult characteristics and effectiveness/patient satisfaction variables. To this end, variables were dichotomized: Q1 and Q3=“Strongly Agree” and “Agree” vs. Others; Q2=“About the Same”, “Somewhat Better”, and “Much Better” vs. Others; and Q4=“Good” and “Very Good” vs. Others. The effect size was evaluated by Cramer’s *V*.

All analyses were performed with R Package (version 4.3.1). The significance level was set at *p* ≤.05, two-tailed.

## 3 Results

From a total of 2,755 patient interviews, 96.1% reported being satisfied, stating that they had a good or excellent experience with telerehabilitation. This result was seen with both new and follow-up patients. From the perspective of rehabilitation professionals, 92.9% rated their experience as “good” or “very good”, also indicating strong satisfaction with the modality, but not as robust as the patients’ ratings (Table 1).

**Table 1:** Patients’ overall satisfaction with telerehabilitation

		Overall experience with telerehabilitation					Pearson’s Chi-squared test^*^				
	*N*	Very poor	Poor	Acceptable	Good	Very good	Satisfaction	Chi	DF	*p*	*Cramer*’*s V*
Perspective
Patient	2,755	0.2%	0.4%	3.3%	30.6%	65.5%	96.1%	11.7	1	.001	.06
Rehabilitation practitioners	668	0.0%	0.1%	6.9%	62.1%	30.8%	92.9%
Appointment
First consult	322	0.0%	0.9%	4.3%	31.7%	63.0%	94.7%	1.5	1	.220	.03
Follow-up	2,433	0.2%	0.3%	3.2%	30.5%	65.8%	96.3%

^*^ “Good” and “Very good” VS Others.

When asked to compare remote treatment with in-person care, 85.1% of the patients said that telemedicine was equal to or better than in-person consults, with no statistically significant difference between first consult and follow-up appointments. Among the providers, however, this percentage fell to 39.7% (Table 2).

**Table 2:** Comparison between telerehabilitation and in-person consultation

		Compared with in-person consultations, how would you evaluate the telerehabilitation session?					Pearson’s Chi-squared test^*^				
	N	Much worse	Somewhat worse	About the same	Somewhat better	Much better	About the same or better	Chi	DF	*p*	*Cramer*’*s V*
Perspective
Patient	2,755	1.9%	13.0%	51.3%	12.2%	21.6%	85.1%	605.7	1	<.001	.42
Rehabilitation practitioners	668	7.6%	52.5%	27.8%	9.4%	2.5%	39.7%
Appointment
First consult	322	2.2%	15.8%	37.9%	19.6%	24.5%	82.0%	2.5	1	.115	.03
Follow-up	2,433	1.8%	12.7%	53.1%	11.2%	21.2%	85.5%

^*^“About the same”, “Somewhat better” and “Much better” VS Others.

The telemedicine system was considered effective by 98.2% of the patients, who agreed (or strongly agreed) that the rehabilitation practitioner had successfully listened to them, answered questions, cleared up doubts, and provided pertinent guidance and instructions. At their first consults, 96.9% of the patients attested to the effectiveness of telerehabilitation. This percentage was not statistically different for existing patients. Effectiveness was ratified by 98.7% of rehabilitation practitioners (Table 3).

**Table 3:** Assessment of telerehabilitation effectiveness

		The healthcare practitioner satisfactorily listened to the concerns, offered guidance, and answered the questions during your telerehabilitation session.					Pearson’s Chi-squared test^*^				
	N	Strongly disagree	Disagree	Neutral	Agree	Strongly agree	Effectiveness	Chi	DF	*p*	*Cramer*’*s V*
Perspective
Patient	2,755	0.4%	0.5%	0.9%	4.9%	93.3%	98.2%	0.4	1	.543	.01
Rehabilitation practitioners	668	0.1%	0.7%	0.4%	40.3%	58.4%	98.7%
Appointment
First consult	322	0.9%	0.6%	1.6%	6.2%	90.7%	96.9%	2.9	1	.091	.04
Follow-up	2,433	0.3%	0.5%	0.8%	4.8%	93.6%	98.4%

^*^ “Strongly agree” and “Agree” vs Others.

Of the 322 patients surveyed at their first consultation, 97.2% agreed or strongly agreed that their expectations were met.

In terms of patient satisfaction with interdisciplinary synchronous telemedicine, the results show that the majority rated their experience as good or very good, with no association with other variables (Table 4) or patient’s age (*r_*s*_*=+.01, *n* = 2,755, *p* = .707).

**Table 4:** Evaluation of telerehabilitation according to patient characteristics

Characteristics	N	Satisfaction (a)	Remote vs. in-person (b)	Effectiveness (c)
Sex
Female	1,353	96.6%	85.1%	98.2%
Male	1,402	95.6%	85.1%	98.3%
p-value^*^		.233	1.000	.900
Hospital location (region)
Midwest	1,272	96.1%	84.5%	98.2%
Southeast	807	96.8%	83.5%	97.9%
Northeast	609	95.7%	88.8%	99.2%
North	67	91.0%	80.6%	94.0%
p-value^*^		.121	.021	.015
Team
Medical specialties	1,368	96.2%	83.5%	97.9%
Paramedical specialties	1,387	96.0%	86.7%	98.6%
p-value^*^		.901	.022	.229
Diagnostic group
Brain injury	1,363	96.1%	84.4%	98.4%
Orthopedics	602	96.2%	87.9%	98.7%
Spinal cord injury	590	95.6%	85.8%	97.3%
Rheumatology	69	98.6%	75.4%	100.0%
Oncology	29	100.0%	93.1%	100.0%
Other diagnoses	102	96.1%	78.4%	97.1%
p-value^*^		.745	.012	.268

(a) Percentage of good or very good; (b) Percentage of about the same or better; (c) Percentage of agree of strongly agree. ^*^p-value by Pearson’s Chi-squared test.

Overall satisfaction scores were similar across sex, hospital location, practitioner specialty, and patient’s diagnoses; in other words, this positive assessment was not linked to any of the aforementioned variables, in accordance with Pearson’s Chi-squared test. On the other hand, we noted that geographical location of the hospital influenced telerehabilitation effectiveness scores and comparison with in-person treatment scores; they were lower in the northern regions of Brazil. In terms of comparison between remote and in-person sessions, we saw higher scores from patients treated by a multidisciplinary team compared to lower scores from patients with rheumatological and other diagnoses (Table 4).

**Fig. 1:**
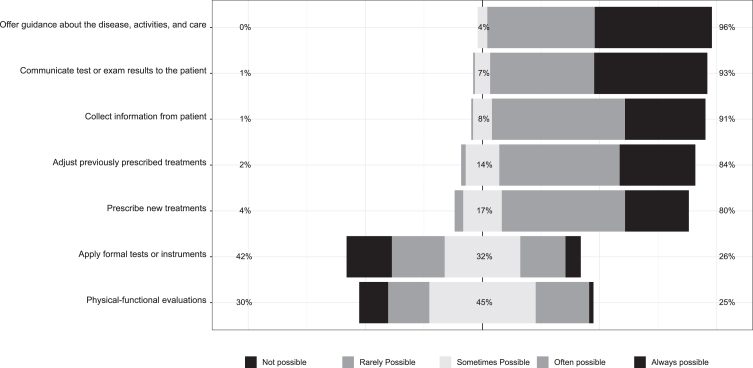
Medical procedure during telerehabilitation.

Healthcare professionals also assessed the viability of certain procedures during telerehabilitation. For them, it was often or always possible to collect information (91%); communicate test or exam results to the patient (93%); and offer guidance about the disease, activities, and care (96%). A slightly smaller percentage of practitioners found it often or always possible to prescribe new treatments (80%) and adjust previously prescribed treatments (84%). Only 26% of providers reported that it was viable (often or always possible) to use formal tests or instruments, while 25% felt it was often or always possible to run physical-functional evaluations remotely (Fig. 1). In sum, 95% of practitioners reported that telerehabilitation often or always contributes to the treatment of patients.

**Fig. 2:**
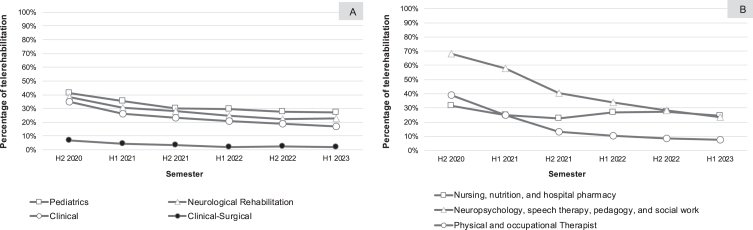
Telerehabilitation percentage in medical (2A) and paramedical (2B) specialties.

As the pandemic subsides, the use of telemedicine has fallen from 37% in the second half of 2020 to 14% in the first half of 2023. We saw a considerable decrease in neuropsychology, speech therapy, pedagogy, and social work, which dropped from 68% at the height of the pandemic, to 23% in the first half of 2023. The decrease in telemedicine was even greater in the field of motor rehabilitation (physical and occupational therapy), dropping from 39% in the second half of 2020, to 7% in the first half of 2023. On the other hand, care-delivery providers, such as nurses and nutritionists, maintained the same level of remote sessions, varying between 31% in 2020 and 24% in 2023 (Fig. 2).

Non-surgical medical specialties also began using telemedicine for monitoring and treating their patients at the start of the pandemic, with clinical subspecialties (genetics, clinical medicine, geriatrics, urology, and others) using telemedicine 35% of the time; neurological rehabilitation (brain and spinal cord injury) 38%; and pediatric rehabilitation using remote sessions 41% of the time. There was a decline in telemedicine consults in all these fields, dropping to 17%, 23% and 27% of the time, respectively. Surgical specialties (neurosurgery and orthopedics) benefitted little from telemedicine, with use of this modality at 7% in the second half of 2020, and at 2% in the first half of 2023 (Fig. 2).

## 4 Discussion

The need for continuous follow-up and management of individuals with disabilities who carry multiple COVID-19 risk factors has made remote delivery of health services during the virus outbreak a major challenge for the interdisciplinary medical teams who care for this population (Chaler et al., 2020; López-Dolado & Gil-Agudo, 2020; Veerapandiyan et al., 2020). We analyzed how effective the patients and providers thought synchronous telerehabilitation was, as well as their degree of satisfaction with it. We also addressed how telemedicine became an established rehabilitation resource three years after the start of the pandemic.

The results show that most surveyed patients, caregivers and professionals rated their experience with remote care as effective, and the practitioners as successful in answering their questions and providing pertinent guidance and information. This result was similar across diagnoses, practitioner specialty, hospital location, age and sex. No differences were observed in the scores of the patients’ first consult, compared to their scores on follow-up. Almost all the first consult patients reported that their expectations were met. Most of the patients rated their remote sessions as good or better than in-person treatment, unlike the practitioners, who mostly preferred in-person sessions. These outcomes point to the effectiveness of synchronous telerehabilitation in delivering care and satisfaction during the COVID-19 outbreak, validating previous experiences and recent studies on telerehabilitation (Cacciante et al., 2022; Killingback et al., 2023; Muñoz-Tomás et al., 2023).

Randomized telerehabilitation trials showed evidence that these remote services are equivalent to in-person sessions for patients with stroke (Laver et al., 2020; Tchero et al., 2018). Later studies conducted during the pandemic, such as the one by Tenforde et al. (2020), reported that telerehabilitation in sports was classified as “excellent” or “very good” by both patients and practitioners. Bhuva et al. (2020) noted that the majority of patients in a rehabilitation and physical medicine clinic preferred telemedicine consults to in-person sessions. Remote neurology consults were deemed on par with regular outpatient visits, and considered more efficient (Iodice et al., 2021), shining a more favorable light on telerehabilitation (Aquino et al., 2021). A study by Heiskanen et al. (2021) noted that after their experiences with telerehabilitation, almost half of the providers from different rehabilitation specialties planned to use remote medicine regularly after the pandemic. A systematic review by Alonazi (2021) on pediatric physical therapy during the COVID-19 pandemic reported that all the studies they reviewed noted that telerehabilitation had a positive impact on different clinical conditions. The studies also revealed that both the rehabilitation providers and the children’s parents or caregivers were satisfied with the remote delivery of rehabilitation services. Curiously, the addition of more weekly sessions for aphasia and neurorehabilitation was well received, compared to traditional in-person provision of these same treatment services (Capra & Mattioli, 2020).

The review by Kairy et al. (2009) showed that patients had higher levels of satisfaction with tele-rehabilitation than the practitioners. In our own study, when we compared the answers given by the patients with the healthcare providers’ responses, we see that both rate their experiences with telerehabilitation as “good” or “very good”. However, when we assess only the percentage that answered “very good”, we see an important difference between the evaluation by patients (65.5%) and the assessment of the practitioners (30.8%). We observed the same phenomenon when both were surveyed about the effectiveness of telerehabilitation, in which 93.3% of the patients strongly agreed that during telerehabilitation sessions, they were able to share their complaints, receive guidance and feedback, and have their doubts addressed. Among the rehabilitation providers, the “strongly agree” percentage was only 58.4%.

This critical gaze by the rehabilitation professionals is also evident in how they answered questions about procedures during telehealth sessions: only one-fourth of the surveyed practitioners judged it feasible to apply formal tests and instruments or conduct physical-functional evaluations remotely. Studies by Hewitt et al. (2020) and Hewitt & Loring (2020) already highlighted the challenge of neuropsychological assessment via telehealth consults. Miner (2021) reported that the continuation of post-pandemic telemedicine might require adaptability on the part of practitioners in learning to perform physical examinations remotely. In this sense, Wahezi et al. (2020), for example, proposed techniques for musculoskeletal and neurological examinations that can be used effectively in telemedicine. It is important to stress that, despite differences between practitioner and patient assessments in general, providers in this study affirmed that telerehabilitation contributed to patients’ treatment. This result corroborates findings by Buabbas et al. (2022), in that most providers they surveyed reported benefits of telerehabilitation and said they were in favor of continuing to use telerehabilitation to support conventional physical therapy care, despite the lack of technologicalinfrastructure.

Technological advances over the last few decades have contributed to the telehealth model, such as the emergence of new devices and biosensors that permit remote monitoring of patients, and the development of self-administered tests like the Multiple Sclerosis Performance Test (MSPT), which includes data obtained via gyroscope and accelerometer (Xiang & Bernard, 2021). Furthermore, the pandemic significantly increased the use of videoconferencing tools for various professional activities. In this sense, we noted a positive transformation brought about by innovative solutions in the healthcare sector, with the aim of mitigating the impact of COVID-19 on human health (Doraiswamy et al., 2020).

In Brazil, telemedicine was regulated throughout the pandemic, on a provisional emergent basis (Morosini, 2021). The sudden onset of this modality raised ethical and legal questions associated with the practice of telehealth services, which still lacks extensive regulations specific to guaranteeing equitable access, quality services, sustainable costs, professional responsibility, respect for patient’s privacy, data protection, and confidentiality (Solimini et al., 2021). Furthermore, we urgently need a global consensus on definitions, limits, protocols, monitoring, evaluation, and data privacy (Doraiswamy et al., 2020).

Three years after the start of the pandemic, there remains a significant percentage of rehabilitation subspecialties that still use telemedicine, which suggests that telerehabilitation has been established as an additional resource, joining traditional in-person sessions. The use of telerehabilitation is directly associated with the type of treatment that the patient will be offered. In specialties like nursing and nutrition counseling, 24% of their consults remain remote. These sessions are focused on monitoring the patient’s state of health and on providing guidance for self-care. Neuropsychology also continues to deliver a portion of their sessions via telemedicine, with consults focused on psychoeducational groups and providing individual recommendations. The physical rehabilitation teams, however, practically abandoned the use of telemedicine since this model of delivery significantly limits the primary activities of these providers. Fernandes et al. (2022) reported that practitioners and patients alike expressed concern about conducting physical therapy sessions online. Clinical and rehabilitation subspecialties continue to offer between 17% and 27% of their sessions online. This represents a significant percentage of their consults. Surgical specialties, on the other hand, do not avail themselves of this delivery mode due to the nature of the treatment involved, and limit telemedicine to occasional sessions for post-operative instructions and recommendations.

This study has some limitations. First, most of the participants were already patients of the SARAH Network, which facilitates the transition from in-person consults to telemedicine sessions, since they had already been evaluated at the SARAH hospital. We did not, however, find any statistically significant difference in the scores of new patients versus existing patients. Second, the effectiveness of telerehabilitation was evaluated only within the scope of a successful session; we did not assess the impact of proposed interventions nor adhesion to prescribed treatments. On the other hand, even if subjectively, the practitioners in this study reported that telerehabilitation positively affected the patients’ treatment. Finally, it is important to note that the satisfaction and effectiveness of telerehabilitation were assessed within the context of a pandemic when most of the population was in social isolation and this modality emerged as the only solution to continue the rehabilitation process. Future studies are needed to evaluate whether the level of satisfaction with telerehabilitation will remain the same post-pandemic.

Nevertheless, the findings indicate alternative options in healthcare delivery for the future, specifically new means of providing continuous follow-up care to a population that requires life-long management. Perhaps in the post-COVID-19 era, healthcare will incorporate teleconsult modalities into rehabilitation and medical protocols, resulting in more cost-effective allocation of funding and human resources, and less strain and travel among patients who must routinely submit to ongoing medical consults and treatment. Studies already point to the economic advantages of using telerehabilitation as an additional treatment modality (Baffert et al., 2023; Grigorovich et al., 2022), including in countries with low- to mid-socioeconomic status (Nizeyimana et al., 2022).

## 5 Conclusion

Our study shows that the use of telerehabilitation can be an effective resource for treating various rehabilitation pathologies. Most patients, caregivers, and healthcare providers classified their experience with remote care during the pandemic as effective. There were no differences in assessment scores between new patients and existing patients. Almost all the new patients reported that their expectations were met with telerehabilitation. A majority of patients rated remote care as good as or better than in-person treatment, unlike the providers, the vast majority of which rated in-person consults as better. The social isolation caused by the pandemic rendered telerehabilitation a viable and effective alternative for monitoring rehabilitation patients; it also helped forge a favorable view of this treatment modality. Healthcare professionals from various fields rate in-person consults as more effective, likely because remote sessions do not lend themselves to accuracy in clinical, neurological, and neuropsychological evaluations. Three years after the start of the pandemic, even with a return to in-person treatment, we see a telerehabilitation culture growing, as a means of supplementing the in-person rehabilitation process, with great potential for establishing this model of treatment in a network of rehabilitation hospitals.

## Conflict of interest

The authors of this manuscript declare that they have no relevant financial or personal relationships with individuals or commercial interests (entities producing, marketing, re-selling, or distributing health care goods or services consumed by, or used on, patients) that could inappropriately influence or bias their work.

## Supplementary Material

Supplementary Material
