# Supplementary material for: Telerehabilitation from the perspective of patients and healthcare providers: A 3-year follow-up study
Source: NeuroRehabilitation. 2024 Aug 29;55(1):103–15. doi: 10.3233/NRE-230385 (PMC11380237; doi:10.3233/NRE-230385)
Supplement: Supplementary Material [file nre-55-nre230385-s001.docx]

**Supplementary Material**

**Supplementary Material 1. Patients' Survey: SMS Invitation.**

SARAH NETWORK: Would you like to take part in our satisfaction survey regarding the care received through telemedicine? Thank you for your cooperation. <http://www.sarah.br/pesquisa?12s3rff43>

**Supplementary Material 2. Patient Survey Questionnaire.**

*Instructions*

*Due to the COVID-19 pandemic, and in compliance with the recommendations of public health authorities, for your safety, our team has been conducting patient follow-ups through teleconsultations. We would like to assess your experience with this new form of care!*

*Question 1*

*The healthcare practitioner satisfactorily listened to the concerns, offered guidance, and answered the questions during your telerehabilitation session.*

| *Strongly disagree* | *Disagree* | *Neutral* | *Agree* | *Strongly agree* |
| --- | --- | --- | --- | --- |
| 🞎 | 🞎 | 🞎 | 🞎 | 🞎 |

*Question 2*

*Compared with in-person consultations, how would you rate the telerehabilitation session?*

| *Much worse* | *Somewhat worse* | *About the same* | *Somewhat better* | *Much better* |
| --- | --- | --- | --- | --- |
| 🞎 | 🞎 | 🞎 | 🞎 | 🞎 |

*Question 3*

*Your expectations were met. (New patients only.)*

| *Strongly disagree* | *Disagree* | *Neutral* | *Agree* | *Strongly agree* |
| --- | --- | --- | --- | --- |
| 🞎 | 🞎 | 🞎 | 🞎 | 🞎 |

*Question 4*

*Overall, how was your experience with the teleconsultation?*

| *Very poor* | *Poor* | *Acceptable* | *Good* | *Very good* |
| --- | --- | --- | --- | --- |
| 🞎 | 🞎 | 🞎 | 🞎 | 🞎 |

**Supplementary Material 3. Practitioners’ Survey: SMS Invitation.**

TELEREHABILITATION: We would like to assess your experience with this new form of care! <http://www.sarah.br/pesquisa?12s3rff43>

**Supplementary Material 4. Practitioner Survey Questionnaire.**

*Instructions*

*Please evaluate the following items based on the teleconsultations conducted in recent months.*

*Question 1*

*I satisfactorily listened to the patients' concerns, offered guidance, and answered their questions during the telerehabilitation session.*

| *Strongly disagree* | *Disagree* | *Neutral* | *Agree* | *Strongly agree* |
| --- | --- | --- | --- | --- |
| 🞎 | 🞎 | 🞎 | 🞎 | 🞎 |

*Question 2*

*Compared with in-person consultations, how would you rate the telerehabilitation session?*

| *Much worse* | *Somewhat worse* | *About the same* | *Somewhat better* | *Much better* |
| --- | --- | --- | --- | --- |
| 🞎 | 🞎 | 🞎 | 🞎 | 🞎 |

*Question 3*

*Overall, how was your experience with teleconsultation?*

| *Very poor* | *Poor* | *Acceptable* | *Good* | *Very good* |
| --- | --- | --- | --- | --- |
| 🞎 | 🞎 | 🞎 | 🞎 | 🞎 |

*Question 4*

*Do you believe that the teleconsultations you conducted contributed to the patient's treatment?*

| *Never* | *Rarely* | *Sometimes* | *Often* | *Always* |
| --- | --- | --- | --- | --- |
| 🞎 | 🞎 | 🞎 | 🞎 | 🞎 |

*Question 5*

*Considering the procedures and medical activities that you practice, please evaluate how feasible it was to carry them out during the teleconsultation.*

|  | *Not possible* | *Rarely possible* | *Sometimes possible* | *Often possible* | *Always possible* |
| --- | --- | --- | --- | --- | --- |
| *Collect information from patient* | 🞎 | 🞎 | 🞎 | 🞎 | 🞎 |
| *Physical-functional evaluations* | 🞎 | 🞎 | 🞎 | 🞎 | 🞎 |
| *Apply formal tests or instruments* | 🞎 | 🞎 | 🞎 | 🞎 | 🞎 |
| *Offer guidance about the disease, activities, and care* | 🞎 | 🞎 | 🞎 | 🞎 | 🞎 |
| *Communicate test or exam results to the patient* | 🞎 | 🞎 | 🞎 | 🞎 | 🞎 |
| *Prescribe new treatments* | 🞎 | 🞎 | 🞎 | 🞎 | 🞎 |
| *Adjust previously prescribed treatments* | 🞎 | 🞎 | 🞎 | 🞎 | 🞎 |
